# Significant barriers to diagnosis and management of adrenal insufficiency in Africa

**DOI:** 10.1530/EC-20-0129

**Published:** 2020-04-28

**Authors:** Thabiso R P Mofokeng, Salem A Beshyah, Fazleh Mahomed, Kwazi C Z Ndlovu, Ian L Ross

**Affiliations:** 1 Department of Medicine, University of the Free State, Bloemfontein, South Africa; 2 Department of Medicine, Dubai Medical College, Duabi, United Arab Emirates; 3 Department of Endocrinology, Mediclinic Airport Road Hospital, Abu Dhabi, United Arab Emirates; 4 Division of Endocrinology, Department of Medicine, University of Cape Town, Cape Town, South Africa

**Keywords:** adrenal insufficiency, hypoadrenalism, Sub-Saharan Africa, Middle East and North Africa, s-cortisol serum cortisol

## Abstract

**Background:**

The burden and management of primary adrenal insufficiency (PAI) in Africa have not been well documented. We aimed to identify specific disease characteristics, patient demographics, and patterns of clinical management in established PAI in Africa.

**Methods:**

An online survey of physicians’ experience relating to PAI.

**Results:**

There were 1334 responses received, 589 were complete, and 332 respondents reported managing patients with hypoadrenalism. The described responses were related to a calculated pool of 5787 patients with hypoadrenalism (2746 females, 3041 males), of whom 2302 had PAI. The likely causes of PAI in Sub-Saharan Africa (SSA) vs the Middle East and North Africa (MENA) regions included autoimmune disease (20% vs 60.3%; *P* < 0.001), tuberculosis (34% vs 4.1%; *P* < 0.001), AIDS (29.8% vs 1%; *P* < 0.001), malignancy, and genetic conditions. Sixteen percent of AD patients (376/2302) presented in an adrenal crisis. Medical emergency identification was not used by 1233 (83.6%) SSA vs 330 (40.4%) MENA patients (*P* < 0.001), respectively. Relative non-availability of diagnostic tests across both regions included adrenal antibodies 63% vs 69.6% (*P* = 0.328), s-cortisol 49.4 % vs 26.7% (*P* = 0.004), s-ACTH 55.7% vs 53.3% (*P* = 0.217), and adrenal CT scans 52.4% vs 31.8% (*P* = 0.017) in the SSA and MENA region, respectively. Across the entire cohort, the overall hydrocortisone use and extrapolated proportion of synacthen use were 59.4% and 50.7%, respectively.

**Conclusions:**

Through the perception and practice of healthcare professionals, we identified significant challenges in the diagnosis and management of PAI which may herald high mortality. Differences between regions may reflect the allocation of healthcare resources.

## Introduction

Primary adrenal insufficiency (PAI) is a rare, potentially fatal, but treatable disease (1, 2). The estimated prevalence of PAI in South Africa is three per million (3), in contrast to Western countries in which prevalence estimates vary from 30 per million to 221 per million (4, 5). In contrast, the prevalence of PAI in most of Sub-Saharan Africa (SSA) and the Middle East and North African (MENA) countries is largely unknown.

In Western countries, most cases of PAI are caused by autoimmune destruction of the adrenal cortex (6), followed by malignancy, tuberculosis (TB), human immunodeficiency virus (HIV), and genetic causes such as adrenoleukodystrophy (ALD) (6, 7). Prevalence data on PAI in SSA have been inferred from case reports and small studies examining adrenal insufficiency in TB patients (8). There is an expectation that the aetiology of PAI in SSA is considerably influenced by the heavy burden of HIV (9) and TB (10, 11, 12) in SSA, but this has never been confirmed. In contrast, since the burden of HIV and TB in the MENA region and Europe are low (13, 14), PAI attributable to these conditions is likely to be low.

The diagnosis of PAI is often delayed by between 3 and 6 months (15). It is challenging due to the insidious onset of non-specific symptoms such as weakness, fatigue, musculoskeletal pain, weight loss, abdominal pain, depression, and anxiety over months or years (16). Adrenal insufficiency is managed with the combination of glucocorticoids and mineralocorticoids, which are life-saving. Despite the availability of appropriate replacement therapy in developed countries, there is a residual increased mortality, morbidity, and reduced quality of life (12) associated with PAI (17, 18) in the European populations. Morbidity, mortality, quality of life, and availability of replacement therapy in PAI, specifically, in SSA and MENA regions are mostly unknown.

Comprehensive healthcare is not provided for many inhabitants of Africa for a variety of financial and governance challenges, typical of other low and middle-income countries (LMIC) (19, 20, 21, 22, 23). The annual government health spending per capita in SSA ranges from $942 (South Africa) to $32 in (Democratic Republic of Congo) (24), with a median of $109 for all African countries (25). In contrast, the proportion of GDP health spent in the MENA region was 4.7% ($403 per person) (26), with the allocation to healthcare in Saudi Arabia of $2466 per person in 2014 vs Yemens’ $202 per person (27). Rapid unplanned urbanization exerts pressure on limited resources in SSA, potentially compromising healthcare. Several papers indicate challenges relating to the availability of laboratory tests for PAI (28, 29) and imaging diagnostic tests due to financial constraints in Africa (19, 30, 31). Deficiencies in special investigations may lead to delays in the diagnosis of PAI and treatment with potentially fatal consequences (32).

There is a wide range of doctor to patient ratios in both SSA and the MENA region reflecting different levels of development. The estimated ratios in SSA vary from 1:54,213 in Central Somalia to 1:1639 in South Africa (33), whereas the ratio in MENA is 1.3 per 1000 (26). SSA has the lowest ratings for well-being and the lowest satisfaction with healthcare, compared to the highest scoring non-English-speaking countries of northern Europe (25).

Having recognized the sparse nature of data, we sought to identify challenges in the diagnosis and management of PAI in SSA and MENA region. We attempted to evaluate this through the perceptions and practices of a large sample of physicians in these regions using an online questionnaire. We hypothesized that several barriers will be identified, limiting optimal diagnosis and management. We also hypothesized that, by comparing the SSA to the MENA regions, differences in the diagnosis and management of PAI will be detected, potentially influenced by the level of affluence and the reliance on relatively inexperienced doctors, without the experience of endocrinologists to make a diagnosis of this rare condition.

## Methods

### Setting

This cross-sectional electronic questionnaire-based study was conducted between January and June 2017. For the creation, dissemination, and analysis of the questionnaire, Survey Monkey® (SVMK Inc., San Mateo, CA, USA) was used. The questionnaire was electronically sent to a convenience sample of healthcare professionals primarily residing and practicing in the Middle East and Africa (Supplementary Appendix 1, see section on supplementary materials given at the end of this article). The initial invitation email explained the rationale of the study. Biweekly reminders were sent to enhance the numbers and quality of responses. Repeat submissions from the same internet protocol address were automatically blocked by the survey service. The patients in Africa were identified using a large commercial email database of clinicians in Africa (MedPages, Cape Town, South Africa) and the MENA regions’ physicians were identified from a large professional email database held by the investigators. All respondents provided consent before proceeding with the actual survey questions.

### Research and ethics

Approval to conduct this survey was obtained from the University of Cape Town (UCT) Faculty of Health Sciences Human Research and Ethics Committee.

### The questionnaire

The questionnaire sought to establish the grade, rank, and location of the physicians who manage their patients with PAI. We asked respondents to identify whether they practised in rural or urban regions. The respondents were also asked to estimate the number of their patients with PAI, as well as the diagnostic and management problems that they encountered at the time of the survey. The questionnaire asked the physicians to report patient demographics, aetiology, presentation, therapy, and to comment on the limitations of diagnosis and treatment of PAI (Supplementary Appendix 2). The final English version was translated into French.

### Data analysis

Two types of data were captured. First, data describing the attributes of the respondents were retrieved directly (*n* = 332). Secondly, data characterizing patients were synthesized based on the numbers of patients seen by a given physician and the relative proportions of the physicians’ responses (calculated patients were 5787). However, no direct patients’ data were collected. The results were expressed in numbers and adjusted for any missing responses by expression as a percentage of total responses per individual question. Groups were compared using Chi-squared tests or Fisher’s exact tests when one of the cell counts was below 5. A two-sample test of proportions was used to compare two proportions of subgroups within variables of the combined cohort. All analyses were performed using STATA statistical software, version 15 (StataCorp, College Station, TX, USA). The level of significance was assigned at *P* < 0.05.

## Results

### Characteristics of respondents

A total of 1334 physician responses were received, of whom 589 completed the questionnaire, and of these, 332 respondents confirmed that they, at the time of the survey, manage patients with hypoadrenalism (Supplementary Appendix 1). The respondents were mostly non-endocrine specialists (48.8%) and primary care doctors (33.3%) and the minority (17.6%) were specialist endocrinologists. The proportion who identified themselves as senior doctors in the MENA region was 83.9% (47/56), compared to 62.3% (165/265) in SSA (*P* = 0.002). Regional distribution differed, with 57.1% (32/56) of respondents from MENA self-identified as endocrinologists, compared to 9.8% (27/275) in SSA (*P* < 0.001).

A higher proportion of MENA respondents vs SSA practised in a government clinic (7.1% vs 1.1%, *P* = 0.017) and in hospitals (MENA 42.9% vs SSA 28.6%, *P* = 0.036), respectively. The proportion of private clinical practice was higher in SSA vs the MENA region (28.6% vs 7.14%, *P* < 0.001). Overall, in this survey, most respondents 87% practised in cities, but relatively more respondents in SSA 14.5%, compared to MENA 1.8% (*P* = 0.006) practised in rural regions (Table 1).

**Table 1:** Professional and practice profiles of medical practitioners (respondents) who participated in survey.

Attributes	SSA^a^ (*n* = 276)	MENA^a^ (*n* = 56)	*P*-value^b^	Total (%, 95% CI)	*P*-value^c^
**Grade (experience)**					
Senior	165 (62.3)	47 (83.9)	0.002	212 (66.0, 60.6–71.0)	Reference
Middle grade	78 (29.4)	7 (12.5)	0.009	85 (26.5, 21.9–31.6	<0.001
Junior	22 (8.3)	2 (3.6)	0.276	24 (7.5, 5.0–10.9)	<0.001
**Type of practice**					
Government hospital	79 (28.6)	24 (42.9)	0.036	103 (31.0, 26.2–36.2)	Reference
Government clinic	3 (1.1)	4 (7.1)	0.017	7 (2.1, 1.0–4.4)	<0.001
Private hospital	69 (25.0)	14 (25.0)	1.000	83 (25.0, 20.6–30.0)	0.084
Private clinic	79 (28.6)	4 (7.14)	<0.001	83 (25.0, 20.6–30.0)	0.084
University based	46 (16.7)	10 (17.9)	0.828	56 (16.9, 13.2–21.3)	<0.001
**Medical speciality**					
Endocrinologist	27 (9.8)	32 (57.1)	<0.001	59 (17.8, 14.0–22.4)	Reference
General practitioner	107 (38.9)	3 (5.4)	<0.001	110 (33.2, 28.3–38.5)	<0.001
Non-endocrine specialist	141 (51.3)	21 (37.5)	0.060	162 (48.9, 43.6–54.3)	<0.001
**Practice locality**					
Urban	236 (85.5)	54 (98.2)		291 (87.6, 83.6–90.8)	Reference
Rural	40 (14.5)	1 (1.8)	0.006	41 (12.4, 9.2–16.4)	<0.001
**Experience managing adrenal crisis**					
No patients	154 (55.8)	25 (45.4)	0.160	179 (54.1, 48.6–59.4)	Reference
1 patient	76 (27.5)	16 (29.1)	0.814	92 (27.8, 23.2–32.9)	<0.001
2–5 patients	41 (14.9)	8 (14.6)	0.953	49 (14.8, 11.4–19.1)	<0.001
>5 patients	5 (1.81)	6 (10.9)	0.004	11 (3.3, 1.8–5.9)	<0.001

^a^Data are presented as number (percentage); ^b^*P*-value: comparison between SSA and MENA; ^c^*P*-value: comparison across the entire group of medical practitioners who participated in the survey.

MENA: Middle East and North Africa; SSA: Sub-Saharan Africa.

When medical practitioners were compared across the entire cohort, there was a preponderance of senior ranked physicians, a predominance of government-employed medical practitioners, the minority were endocrinologists, and a substantial proportion practised in urban vs rural settings. The majority of practitioners had no experience managing an adrenal crisis (Table 1).

#### Patients characteristics

The physicians’ responses were based on their experiences of 5787 patients with hypoadrenalism. Adrenocorticotropic hormone (ACTH) deficiency disease, long term steroid use, and adrenalectomy accounted for 1275, 1896, and 314 patients, respectively, and were not included in the analysis of patients with PAI. Of these, the remainder constituted 2302 (39.8%) patients who had PAI, with 1144 males (49.7%) and females 1158 (50.3%). The aetiology of PAI was considered autoimmune in the majority of patients from the MENA region (60.3%), compared with 20% in the SSA region, *P* < 0.001. While the proportion of PAI attributable to TB in SSA was higher at 34% compared to 23% in MENA (*P* < 0.001), similarly, PAI attributable to Acquired Immune Deficiency Syndrome (AIDS) was higher in SSA at 29.8%, compared to 4.1% in MENA (*P* < 0.001). In the entire group of PAI patients, the aetiology was considered to be TB in 535 (23%), AIDS 447 (19%), malignancy 96 (4%), and genetic causes including ALD 106 (5%) (Table 2).

**Table 2:** Reported characteristics of adrenal insufficiency patients under the care of the responding physicians.

Patients’ characteristics	SSA^e^ (*n* = 2885)	MENA^e^ (*n* = 2902)	*P*-value^f^	Total (%, 95% CI)	*P*-value^g^
**Causes of hypoadrenalism**					
Bilateral adrenalectomy	239 (8.3)	75 (2.6)	<0.001	314 (5.4, 4.9–6.0)	<0.001^d^
ACTH deficiency disease	595 (20.6)	680 (23.4)	0.010	1275 (22.0, 21.0–23.1)	<0.001^d^
Long term steroid use	577 (20.0)	1319 (45.4)	<0.001	1896 (32.8, 31.6–34.0)	<0.001^d^
Primary adrenal insufficiency	1474 (51.1)	828 (28.5)	<0.001	2302 (39.8, 38.5–41.0)	
**Age and sex distribution of primary adrenal insufficiency**					
Sex (females)^a^	779 (52.8)	379 (45.8)	0.001	1158 (50.3, 48.2–52.4)	
Age 0–15	101 (6.8)	41 (5.0)	0.069	142 (6.2, 5.2–7.2)	<0.001
Age 16–60	1 235 (83.8)	539 (65.1)	<0.001	1774 (77.1, 75.2–78.8)	Reference
Age >60	138 (9.4)	248 (30.0)	<0.001	386 (16.7, 15.3–18.4)	<0.001
**Aetiology of primary adrenal insufficiency**^b^					
Autoimmune	294 (20.0)	499 (60.3)	<0.001	793 (34.4, 32.5–36.4)	Reference
Tuberculosis	501 (34.0)	34 (4.1)	<0.001	535 (23.2, 21.5–25.0)	<0.001
ALD and genetic disorders	74 (6.9)	32 (3.9)	0.003	106 (4.6, 3.8–5.5)	<0.001
AIDS	439 (29.8)	8 (1.0)	<0.001	447 (19.4, 17.8–21.1)	<0.001
Malignancy	79 (5.4)	17 (2.1)	<0.001	96 (4.2, 3.4–18.4)	<0.001
Other	54 (3.7)	22 (2.7)	0.195	386 (16.7, 15.3–5.1)	<0.001
**Auto immune associations**^b^					
Type 1 diabetes	347 (23.5)	95 (11.5)	<0.001	442 (19.2, 17.6–20.9)	Reference
Hypothyroidism	301 (20.4)	137 (16.6)	0.023	438 (19.0, 17.4–20.7)	0.863
Graves’ disease	67 (4.6)	36 (4.4)	0.826	103 (4.5, 3.7–5.4)	<0.001
Pernicious anaemia	123 (8.3)	64 (7.7)	0.604	187 (8.1, 7.0–9.3)	<0.001
Premature ovarian insufficiency	86 (5.8)	57 (6.9)	0.317	143 (6.2, 5.3–7.3)	<0.001

^a^Gender of patients with hypoadrenalism was declared for 2002 patients in SSA and 2736 in MENA; ^b^Percentages calculated out of patients with Addison’s disease (*n* = 1474 for SSA and 828 for MENA); ^c^Percentages of common presenting features according to respondent doctors (*n* = 276 for SSA and 56 for MENA); ^d^Secondary hypoadrenalism causes compared to Addison’s disease; ^e^Results are expressed as number(percentage); ^f^*P*-value: comparison of clinical characteristics of patients between SSA and MENA; ^g^*P*-value: comparison of all the patients in the entire cohort.

AIDS: acquired immunodeficiency syndrome; ALD: adrenoleukodystrophy; MENA: Middle East and North Africa; SSA: Sub-Saharan Africa; TB: *Mycobacterium tuberculosis*.

Autoimmune conditions associated with PAI in descending order of frequency were primary hypothyroidism 438 (19%), type 1 diabetes mellitus 332 (14%), pernicious anaemia 187 (8%), premature ovarian failure 143 (6%), and Graves’ disease 103 (4.5%). There were relatively fewer patients with type 1 diabetes, but relatively more patients with either premature ovarian failure or pernicious anaemia in the MENA region compared with SSA. Eighty-four percent of patients (1926/2302) presented with a constellation of the classical symptoms of hypoadrenalism most notably abdominal pain, nausea, vomiting, diarrhoea, weight loss, and dizziness. In the MENA region, respondents reported a higher proportion of PAI patients presenting with diarrhoea (86% vs 66.2%; *P* < 0.006), abdominal pain (90.4% vs 71.2%; *P* < 0.004) and dizziness (92.4% vs 79.7%; *P* < 0.029), compared to SSA region, respectively. The proportion of patients presenting in an adrenal crisis was similar in both the MENA and SSA regions (16.4% vs 16.2%, respectively; *P* = 0.884). In both geographical regions, a notable proportion of patients (42.6% up to 69.4%) presented with symptoms suggestive of an adrenal crisis (Table 3).

**Table 3:** Reported presenting features and severity of adrenal insufficiency patients under the care of the responding physicians.

Patients’ characteristics	SSA^a^ (*n* = 2885)	MENA^a^ (*n* = 2902)	*P*-value^b^	Total (%, 95% CI)	*P*-value^c^
**Presenting features**^d^					
Gastrointestinal symptoms	227 (82.2)	52 (92.9)	0.047	279 (84.0, 79.6–87.8)	
Nausea	188 (79.7)	46 (86.8)	0.232	234 (81.0, 76.0–85.3)	Reference
Vomiting	174 (73.7)	43 (82.7)	0.175	217 (75.3, 69.9–80.2)	0.076
Diarrhoea	153 (66.2)	43 (86.0)	0.006	196 (69.7, 64.0–75.1)	<0.001
Abdominal pain	168 (71.2)	47 (90.4)	0.004	215 (74.6, 69.2–79.6)	0.047
Anorexia	164 (71.0)	42 (80.8)	0.152	206 (72.8, 67.2–77.9)	0.012
**Non-specific features**					
Dizziness	188 (79.7)	49 (92.4)	0.029	237 (82.0, 77.1–86.3)	Reference
Weight loss	187 (77.3)	47 (88.7)	0.063	234 (79.3, 74.2–83.8)	0.378
Salt craving	116 (50.7)	30 (62.5)	0.135	146 (52.7, 46.6–58.7)	<0.001
Skin pigment	154 (65.8)	40 (75.5)	0.175	194 (67.6, 61.8–73.0)	<0.001
Backache	115 (50.0)	29 (56.9)	0.375	144 (51.3, 45.2–57.2)	<0.001
**Adrenal crisis**	242 (16.4)	134 (16.2)	0.884	376 (16.3, 14.8–17.9)	
**Features suggestive of adrenal crisis**					
Hypoglycaemia	162 (67.8)	40 (76.9)	0.195	202 (69.4, 63.8–74.6)	Reference
Loss of consciousness	97 (42.5)	21 (42.9)	0.968	118 (42.6, 36.7–48.6)	<0.001
Shock	127 (54.3)	29 (55.8)	0.845	156 (54.6, 48.6–60.4)	<0.001

^a^Results are expressed as number (percentage); ^b^*P*-value: comparison of clinical characteristics of patients between SSA and MENA; ^c^*P*-value: comparison of all the patients in the entire cohort; ^d^Percentages of common presenting features according to respondent doctors (*n* = 276 for SSA and 56 for MENA).

When the clinical characteristics of all the patients were compared, notably the vast majority were within the age group of 16 to 60 years. Aetiologically, PAI was attributed predominantly to autoimmune causes, with the most prevalent association being type 1 diabetes mellitus (Table 2). Notably, nausea was the most common presenting gastrointestinal symptom (81%; 95% CI, 76–85%), compared with the other gastrointestinal features. Non-specific clinical features such as dizziness occurred frequently (82.0%; 95% CI, 77.1–86.3%), and in respect of the criteria for an adrenal crisis, hypoglycaemia (69.4%; 95% CI, 63.8–74.6%) was the most common feature (Table 3).

### Diagnostic and management strategies

For the diagnosis of PAI, depending on access and availability, clinicians relied on at least one of the following methods: clinical grounds only, clinical features in addition to serum sodium and serum potassium only, clinical features and serum sodium and/or serum potassium in addition to relevant antibodies, or clinical features in addition to low serum cortisol with synthetic ACTH (tetracosactide) stimulation tests to varying degrees. A significant proportion of the MENA group physicians 94.4% (51/54) used serum sodium, cortisol, and synthetic ACTH stimulation tests for the diagnosis, compared to SSA physicians 77.2% (183/237) *P* = 0.002 (Table 3). In terms of diagnostic trends across the entire cohort, 80.4% (95% CI, 75.4–84.8%) of practitioners used a combination of clinical features of PAI together with the low serum sodium, serum cortisol, and an ACTH stimulation test to confirm the diagnosis, while 56.2% (95% CI, 50.2–62.3%) of the cohort used clinical features and suggestive biochemistry, without the definitive ACTH stimulation test (Table 4).

**Table 4:** Reported physicians’ practices regarding diagnostic trends of patients with adrenal insufficiency including practitioners’ practices and access to diagnostic facilities.

	SSA^e^ (*n* = 276)	MENA^e^ (*n* = 56)	*P*-value^f^	Total (%, 95% CI)	*P*-value^g^
**The use of various basis of diagnosis**^a,b^					
**Clinical features alone**					
Never used alone	98 (44.6)	23 (48.9)	0.696	121 (45.4, 39.2–51.5)	0.040
Sometimes/often/always used alone	121 (55.0)	24 (51.1)		145 (54.3, 48.1–60.4)	
**Clinical features plus serum sodium combination**					
Never used these 2 criteria alone	59 (26.3)	18 (36.7)	0.313	77 (28.2, 22.9–33.9)	<0.001
Sometimes/often/always used	164 (73.2)	31 (63.3)		195 (71.4, 65.7–76.7)	<0.001^c^
**Clinical features plus serum sodium plus serum cortisol combination**					
Never used these 3 criteria alone	98 (44.0)	20 (42.6)	0.861	118 (43.7, 37.7–49.8)	0.004
Sometimes/often/always used alone	125 (56.0)	27 (57.4)		152 (56.2, 50.2–62.3)	0.658^c^
**Clinical features, low serum sodium plus cortisol plus ACTH test combination**					
Never used these 4 criteria	54 (22.8)	3 (5.6)	0.002	57 (19.6, 15.2–24.7)	<0.001
Sometimes/often/always used	183 (77.2)	51 (94.4)		234 (80.4, 75.4–84.8)	<0.001^c^
**Inadequate diagnostic test availability**^a,b^					
**Diagnostic tests**					
Never unavailable	78 (33.3)	22 (46.8)	0.210	100 (35.6, 30.0–41.5)	<0.001
Sometimes/often unavailable	145 (62.0)	25 (53.2)		170 (60.5, 54.5–66.3)	
**Adrenal antibody**					
Never unavailable	53 (23.0)	12 (26.09)	0.328	65 (23.5, 18.7–29.0)	<0.001
Sometimes/often unavailable	145 (63.0)	32 (69.6)		177 (64.1, 58.2–69.8)	0.381^d^
**Serum cortisol**					
Never unavailable	108 (46.4)	33 (73.3)	0.004	141 (50.7, 44.7–56.7)	0.238
Sometimes/often unavailable	115 (49.4)	12 (26.7)		127 (45.7, 39.7–51.7)	<0.001^d^
**Serum ACTH**					
Never unavailable	91 (38.7)	21 (46.7)	0.217	112 (40.0, 34.2–46.0)	<0.001
Sometimes/often unavailable	131 (55.7)	24 (53.3)		155 (55.3, 49.3–61.3)	0.212
**Adrenal CT scan**					
Never unavailable	99 (42.5)	29 (65.9)	0.017	128 (46.2, 40.2–52.3)	0.494
Sometimes/often unavailable	122 (52.4)	14 (31.8)		136 (49.1, 43.1–55.1)	0.007^d^

^a^‘Often’ and ‘very often’ response combined as ‘often’; ^b^Percentages calculated out of total response given including ‘not sure’ responses; ^c^Positive response (’Sometimes/often/always’) compared with positive response for use of ‘Clinical features’ as a basis for diagnosis; ^d^Positive response (’Sometimes/often/always’) compared with positive response for non-availability of ‘Diagnostic tests’; ^e^Data are shown as numbers (percentage); ^f^*P*-value: comparison of diagnostic strategies in SSA vs MENA; ^g^*P*-value: comparison of diagnostic strategies across the entire cohort of patients.

ACTH: adrenocorticotropic hormone; MENA: Middle East and North Africa; SSA: Sub-Saharan Africa.

### Patient management trends

The proportion of patients using hydrocortisone alone in the MENA region was 34% (780/828), compared to 28% (588/1474); *P* < 0.001) in SSA. Several formulations of glucocorticoid replacement therapy were used, these included hydrocortisone, prednisolone, cortisone acetate, dexamethasone, and betamethasone in decreasing frequency (Table 5). Very low utilization of fludrocortisone use was described overall, but it was significantly lower in the SSA 368 (25%) vs the MENA 460 (55.6%); *P* < 0.001.

**Table 5:** Reported trends in physicians’ practices regarding management of adrenal insufficiency patient and some challenges/barriers to optimal care.

	SSA^c^ (*n* = 1474)	MENA^c^ (*n* = 828)	*P*-value^d^	Total (%, 95% CI)	*P*-value^e^
**Patterns of glucocorticoid replacement**					
**Therapeutic drugs used**					
Hydrocortisone	588 (39.9)	780 (94.2)	<0.001	1368 (59.4, 57.4–61.4)	Reference
Fludrocortisone	368 (25.0)	460 (55.6)	<0.001	828 (36.0, 34.0–38.0)	<0.001
Cortisone acetate	139 (9.4)	29 (3.5)	<0.001	168 (7.3, 6.3–8.4)	<0.001
Prednisone	442 (30.0)	105 (12.7)	<0.001	547 (23.8, 22.0–25.6)	<0.001
Dexamethasone	129 (8.8)	51 (6.2)	0.026	180 (7.8, 6.8–9.0)	<0.001
Betamethasone	40 (2.7)	-	-	40 (1.7, 1.2–2.5)	<0.001
**Method used in drug dose adjustment**					
Adjusted for body weight	141 (60.8)	22 (43.1)	0.0.021	163 (57.6, 51.6–63.4)	Reference
Adjusted for body surface area	22 (9.4)	7 (13.7)	0.349	29 (10.2, 7.0–14.4)	<0.001
Fixed dose for all patients	72 (31.0)	22 (43.1)	0.097	94 (33.2, 27.8–39.0)	<0.001
**Reported drug non-availability**^a,b^					
All medicine					
Never unavailable	111 (47.8)	30 (66.7)	0.049	141 (50.9, 44.8–56.9)	0.172
Sometimes/often unavailable	110 (47.4)	15 (33.3)		125 (45.1, 39.2–51.2)	
Hydrocortisone					
Never unavailable	150 (64.4)	26 (56.5)	0.118	176 (63.1, 57.1–68.8)	<0.001
Sometimes/often unavailable	72 (30.9)	20 (43.5)		92 (33.0, 27.5–38.8)	
Fludrocortisone					
Never unavailable	62 (26.5)	20 (42.6)	0.008	82 (29.2, 23.9–34.9)	<0.001
Sometimes/often unavailable	148 (63.2)	27 (57.4)		175 (62.2, 56.3–68.0)	<0.001^c^
Prednisone					
Never unavailable	186 (81.2)	37 (82.2)	0.798	223 (81.4, 76.3–85.8)	<0.001
Sometimes/often unavailable	31 (13.5)	7 (15.6)		38 (13.8, 10.0–18.5)	<0.001^c^
Dexamethasone					
Never unavailable	127 (55.0)	31 (70.4)	0.121	158 (57.4, 51.4–63.4)	<0.001
Sometimes/often unavailable	89 (39.5)	10 (22.7)		99 (36.0, 30.3–42.0)	0.458^c^
Betamethasone					
Never unavailable	99 (42.9)	28 (65.1)	0.017	127 (46.4, 40.3–52.4)	0.080
Sometimes/often unavailable	98 (42.4)	9 (20.9)		107 (39.0, 33.2–45.1)	0.142^c^
**Additional barriers to management**^a^					
** Method of patient identification**					
None	1233 (83.6)	330 (40.4)	<0.001	1563 (67.9, 65.9–69.8)	Reference
Bracelet	152 (10.3)	56 (6.8)	0.004	208 (9.0, 7.9–10.3)	<0.001
Card	89 (6.0)	442 (53.4)	<0.001	531 (23.1, 21.4–24.8)	<0.001
**Communication-type barriers to management**^a^					
Educational level	110 (48)	28 (64)	0.161	138 (50.5, 44.4–56.6)	Reference
Language issues	79 (34)	8 (17)	0.010	87 (31.1, 25.8–37.0)	<0.001
Cultural issues	91 (40)	17 (37)	0.154	108 (39.5, 33.7–45.6)	0.010

^a^Percentages according to respondent doctors (*n* = 276 for SSA and 56 for MENA); ^b^Percentages calculated out of total responses given including ‘not sure’ responses; ^c^Resported responses as Number (%); ^d^*P*-value^1^: comparison of management strategies in SSA and MENA; ^e^*P*-value^2^: comparison of all patients’ management strategies.

MENA, Middle East and North Africa; SSA, Sub-Saharan Africa.

Over half of the respondents in both regions, 57.6% (163/283), used bodyweight for dose adjustment of glucocorticoids. The relative proportions for dose adjustment by weight were 60.8% (141/232) in SSA vs 43.1% (22/51) in MENA (*P* = 0.021), whereas the body surface area-based dose adjustment in SSA was 8.0% compared to 12.5% in MENA (*P* = 0.274). The proportion of clinicians using fixed doses in SSA was 72(26.1%), compared to 22 in the MENA region (39.3%) (*P* = 0.046) (Table 5).

In cases of emergency, 32.1% (739/2302) patients were using some form of identification, indicating that they have PAI and need glucocorticoids. Of the 208 patients who used a bracelet, 152 (10.3%) were from SSA vs 56 (6.8%) in the MENA region (*P* = 0.004). Of the 531 who used the identification card, SSA had 89 (6.0%) vs 442 (53.4%) in the MENA region; *P* < 0.001, and 84 patients used other forms of identification.

The clinically significant barriers were mainly related to obtaining replacement therapy in SSA (47.4%, 95% CI 41.0–54.3%) vs MENA (33.3%, 95% CI 21.0–48.4%); *P* = 0.049, and unavailable diagnostic testing such as serum cortisol (49.4%, 95% CI 42.9–55.8%) vs (26.7%, 95% CI 15.7–41.6%) *P* = 0.004 and adrenal CT (52.4%, 95% CI 45.9–58.7%) vs (31.8%, 95% CI 19.7–47.1%) *P* = 0.017 in SSA vs MENA, respectively. Across both regions, there was also relative non-availability of serum ACTH and adrenal antibodies (Table 3). The non-availability of fludrocortisone was reported more often in SSA 63.2% than in the MENA region 57.4% (*P* = 0.008). In SSA 34% of respondents reported language as a barrier to management, compared to 17% in MENA (*P* = 0.010) (Table 5).

Across the entire cohort, the preferred mode of glucocorticoid replacement was hydrocortisone, followed by cortisone acetate, prednisone, dexamethasone, and betamethasone, in descending order. A significant proportion did not receive fludrocortisone. The vast majority of patients across the cohort did not use any form of identification and most clinicians in this survey reported glucocorticoid dose adjusted for body weight, in contrast to those adjusted for body surface area or fixed-dose.

## Discussion

This first Africa and Middle East survey of physicians and their experience in treating patients with PAI revealed significant deficiencies in respect of obtaining critical diagnostic tests necessary for making the diagnosis of PAI. These deficiencies include serum cortisol, serum ACTH, adrenal antibodies, adrenal CT scans and synthetic ACTH stimulation tests in both regions, and replacement therapy, with SSA being more affected than the MENA region. Hydrocortisone was the preferred form of glucocorticoid replacement, but fludrocortisone was not universally prescribed. Dosing adjustments of glucocorticoids were largely made based on body weight, rather than body surface area.

Across the entire cohort, there was a preponderance of senior doctors and a greater urban bias of respondents in this survey. The predominance of senior doctors was more pronounced in the MENA region, suggesting a greater reliance on experienced clinicians for managing this complex disorder, in contrast to SSA, where there was a preponderance of junior doctors. In a trauma emergency study, the group of patients treated by a consultant had a significantly better outcome than the group of patients treated by junior doctors. The analysis of the outcome according to the grade of junior doctors suggested a step-wise improvement in the outcome with seniority in the accident and emergency units, thus supporting that an improved outcome is associated with experience and seniority (34). One could speculate that a preponderance of junior doctors in the SSA region could lead to the diagnosis being missed, with potentially fatal consequences. The under-representation of endocrinologists in the SSA cohort is a deficiency that warrants urgent correction through training.

Clinicians in the SSA identified significant barriers in accessing diagnostic tests, particularly serum cortisol and CT scans of the abdomen. Given the discordant reported synacthen use of 80%, we made a presumptive assessment of the proportion of synacthen use based on the reported serum cortisol availability of 50.7% across both regions. These results may have been influenced by the respondents’ expectation that they should have performed the test. In South Africa, in particular, synacthen is not readily available, and as a non-registered drug, it has to be imported with special permission through the Medicines Control Council (35). It is inferred that, where these special investigations are not widely available, the clinicians in both these regions have to diagnose PAI presumptively, with the result that PAI in SSA and MENA may be under-diagnosed with life-threatening consequences. Significant language barriers must have played a role in suboptimal management of PAI in SSA, compared to the MENA region, considering that for most SSA countries language heterogeneity is the norm (2).

The suboptimal management of PAI is also compounded by a relatively widespread lack of available medicines such as hydrocortisone (36), and fludrocortisone, which poses a clear and present danger to patients, particularly in SSA. Since all glucocorticoids are not equivalent, for example, dexamethasone and prednisolone are considerably more potent than hydrocortisone (37), long term use of prednisolone and dexamethasone has been associated with undesirable side-effects such as wakefulness, nocturia, (38), bone mass reduction (39, 40, 41), hip fractures (42), adverse metabolic health (43, 44), and increased mortality (45). Hydrocortisone dose adjustment is thus critical to mitigating these undesirable side-effects, with weight adjustment in adults being the current preferred mode (43), albeit an imperfect method. Current research efforts to approximate the natural cortisol circadian rhythm through chronotherapy (46) hold the future promise of reduction of the observed side-effects.

A significant proportion of PAI in SSA was suggested as being secondary to underlying AIDS and tuberculosis, whereas in the MENA region these conditions were considered relatively infrequent. The likely explanation for this disparity is the background prevalence of HIV in these two settings (47), which is 0.1% in the MENA region (48), compared to 5% in SSA (49). Given the lower background prevalence of HIV in the MENA compared with SSA regions, it is likely that the combination of abdominal pain and diarrhoea pointed to the diagnosis of PAI, rather than an infective process or HIV.

Consistent with other reports (5, 50, 51), autoimmunity was the major cause across both regions and a predominant cause of PAI in the MENA area compared with SSA. The reported autoimmunity of 20% by the SSA cohort is much lower than was previously found in South Africa, which constituted 50% of the cohort (52). The low autoimmunity detection might be due to the unavailability of specific auto-antibody testing in SSA, and this could also limit the detection of other autoimmune diseases such as autoimmune polyglandular syndromes (APS) (53). The predominance of females in the MENA region to some degree corroborates autoimmunity as the most important underlying aetiology in this region (54). The significantly higher proportion of patients with type 1 diabetes mellitus and hypothyroidism in SSA may reflect a regional experience or bias in which autoimmunity was a predominant cause of PAI in a largely European population (55).

Across both regions, patients presented with a constellation of symptoms, which was similar including salt craving, anorexia, and vomiting. A higher proportion of patients in the MENA region presented with abdominal pain, diarrhoea, weight loss, and dizziness than those in SSA. In a study on the adrenal crisis, patients reported abdominal pain, vomiting, and diarrhoea among the symptoms which led to the adrenal crisis (56). These symptoms may in the future be used as red flags when training patient and family on how to avoid adrenal crises or when to initiate early treatment adjustment. A significant number of patients presented at some stage during their clinical course with an adrenal crisis. Considering the relative predominance of the junior doctors in SSA, it is possible that presenting adrenal crises could either have been missed due to delayed referral to urban centres or non-specific symptoms incorrectly attributable to AIDS by the respondents.

We identified a large proportion of respondents’ patients who did not use some form of medical identification, indicating that they have PAI and need steroids in cases of the emergency. The relative proportions in SSA vs MENA, who did not have any form of medical identification, were 83% and 39%, respectively. In the 1997 North American survey of individuals with PAI, 89.9% of the patients reported wearing Medic Alert bracelets or necklaces. The low proportion of identification use in SSA may represent a surrogate marker of poor patient disease literacy and can contribute to the mortality associated with an adrenal crisis (57). Although there were no significant differences in the proportion of signs suggestive of adrenal crisis such as hypoglycaemia, shock, and loss of consciousness between the two regions, the proportions of signs were higher than the recorded adrenal crises estimated at 16% in both regions. This poor correlation between reported adrenal crises at 16% and signs relating to an adrenal crisis above 40% in both regions suggest possible under-diagnosis of adrenal crises in both regions, to the possible detriment of patients. Considering that the majority of these respondents in both regions had no experience in managing an adrenal crisis, it is possible that many patients are being missed or do not reach the hospital to receive life-saving treatment. We believe that focused Africa-wide doctor, patient, family, and general population training on adrenal crisis prevention, diagnosis, and treatment is warranted.

We postulate that the discrepancies in the available diagnostic and management options for PAI in SSA regions may be due to the per capita gross domestic product (GDP) health-spend, which in 2014 was $2466 in Saudi Arabia according to the WHO (27), as opposed to US $942 (South Africa) to $32 in (Democratic Republic of Congo) (24). Studies have shown that where there are high population densities, there is a tendency toward more physicians per capita (58). This correlates with the urban concentration of physicians in our survey. However, this tendency toward urban concentration detracts from the quality of care in rural settings. This is noteworthy within high per capita health spending countries and even more obvious in low-income countries WHO (27).

The study has strengths and weaknesses that are worth noting: The main limitation stems from the nature of the study being a physicians’ survey rather than a patients series. The survey certainly cannot claim to provide a comprehensive picture of the healthcare of patients with PAI in Africa. The model adopted was based on an electronic survey of the physician’s perceptions of the burden of disease, its characteristics, and challenges. The online survey saves time and effort and allows a large number of physicians to be involved in such a wide area. From these data, we inferred the status of the healthcare levels and barriers. However, a relatively high number of health professionals widely distributed in the three areas have contributed to the data set providing an African perspective of a rare medical condition, contrasting available data from other continents. The use of English and French in the questionnaires allowed more respondents. However, we observed a suboptimal number of available African countries, with respondents from only 18 out of 54 countries. This is in contrast to ten MENA countries, where all countries were represented. Given the electronic nature of the questionnaire, a possible bias toward urban and technically savvy respondents may exist. With internet connectivity mainly in the urban areas in SSA, there may be under-representation of rural health practitioners. Recall bias may influence the numbers of patients and the circumstances of their management. The study is under-representative to generate information on the approximate prevalence of PAI. Moreover, the specific countries were not evaluated individually. Availability of the synacthen test was not probed with a direct question, which represents an omission; hence, we made a presumptive assessment across both regions.

## Conclusion

This is the first Africa-wide and MENA study of primary adrenal insufficiency, demonstrating that there are substantial barriers to diagnosis and management, and these may likely herald high mortality. Fundamental differences exist between Sub-Saharan Africa and the Middle East and North African regions in the options available with respect to diagnosis and management of PAI, with greater availability as a consequence of better health resource allocation. In particular, as PAI is highly treatable, the education of patients and their healthcare providers is paramount. Unfortunately, the deficiencies in the provision of healthcare are not limited to PAI in these regions, but likely represent gross deficiencies for many conditions and require political will to change the course.

## Supplementary Material

Appendix 1. Summary of respondents and their patients’ profiles

Appendix 2. The survey questions with [potential responses] grouped under three different domains.

## Declaration of interest

The authors declare that there is no conflict of interest that could be perceived as prejudicing the impartiality of the research reported.

## Funding

This work did not receive any specific grant from any funding agency in the public, commercial, or not-for-profit sector.

## Compliance with ethical principles

The study was conducted following the ethical principles of the Declaration of Helsinki. Ethical approval was granted by the Faculty of Health Sciences Human Research Ethics Committee, the University of Cape Town, Republic of South Africa (Approval reference HREC/REF: RO42/2016). Informed electronic consent was obtained from all participants before they could proceed to participate in the study.

## Author contribution statement

All the authors contributed to the inception, development, and conduct of the study, including data analysis and drafting and revising of the manuscript for intellectual content. Mofokeng T R P performed questionnaire compilation, data management, analysis, and manuscript development. Beshyah S A performed questionnaire development, data managment, and manuscript development. Mahomed F performed questionaire development. Ndlovu K C Z performed data management and statistical analysis. Ross I L performed conceptualization, manuscript development, and compiled the survey questionnaire. All authors approved the final version of the manuscript.
