# Supplementary material for: Significant barriers to diagnosis and management of adrenal insufficiency in Africa
Source: Endocr Connect. 2020 Apr 28;9(5):445–56. doi: 10.1530/EC-20-0129 (PMC7274557; doi:10.1530/EC-20-0129)
Supplement: Appendix 1. Summary of respondents and their patients’ profiles [file supplementary_table_1.pdf]

Appendix 1. Summary of respondents and their patients' profiles

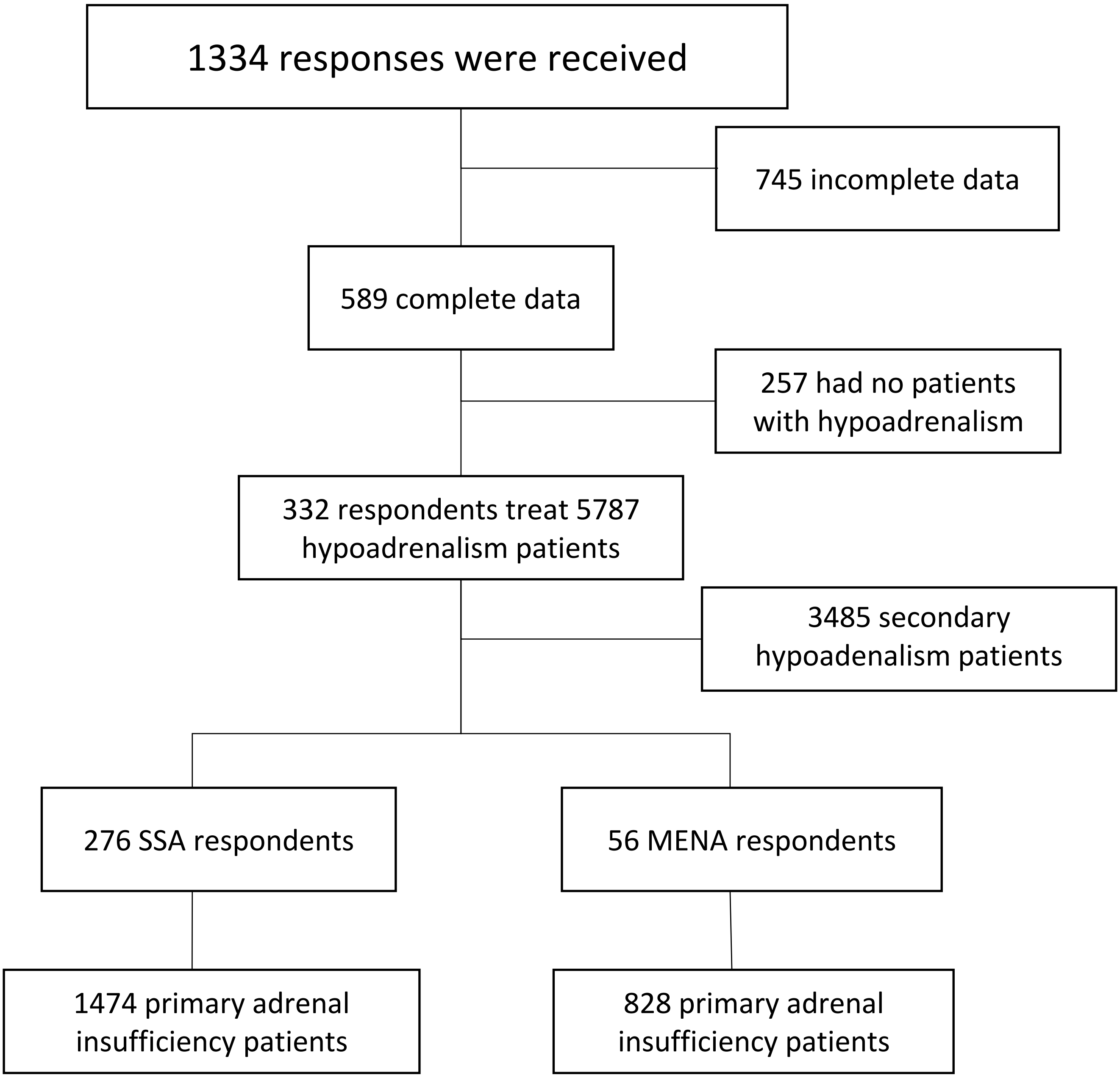

Hypoadrenalism, Primary Adrenal Insufficiency, MENA Middle East North Africa
